# Assessment of peak oxygen uptake during handcycling: Test-retest reliability and comparison of a ramp-incremented and perceptually-regulated exercise test

**DOI:** 10.1371/journal.pone.0181008

**Published:** 2017-07-13

**Authors:** Michael J. Hutchinson, Thomas A. W. Paulson, Roger Eston, Victoria L. Goosey-Tolfrey

**Affiliations:** 1 The Peter Harrison Centre for Disability Sport, School for Sport, Exercise and Health Sciences, Loughborough University, Loughborough, United Kingdom; 2 Alliance for Research in Exercise, Nutrition and Activity, Sansom Institute for Health Research, School of Health Sciences, University of South Australia, Adelaide, Australia; Norwegian University of Science and Technology, NORWAY

## Abstract

**Purpose:**

To examine the reliability of a perceptually-regulated maximal exercise test (PRET_max_) to measure peak oxygen uptake ($\dot{V}O_{2\mathrm{peak}}$) during handcycle exercise and to compare peak responses to those derived from a ramp-incremented protocol (RAMP).

**Methods:**

Twenty recreationally active individuals (14 male, 6 female) completed four trials across a 2-week period, using a randomised, counterbalanced design. Participants completed two RAMP protocols (20 W·min^-1^) in week 1, followed by two PRET_max_ in week 2, or vice versa. The PRET_max_ comprised five, 2-min stages clamped at Ratings of Perceived Exertion (RPE) 11, 13, 15, 17 and 20. Participants changed power output (PO) as often as required to maintain target RPE. Gas exchange variables (oxygen uptake, carbon dioxide production, minute ventilation), heart rate (HR) and PO were collected throughout. Differentiated RPE were collected at the end of each stage throughout trials.

**Results:**

For relative $\dot{V}O_{2\mathrm{peak}}$, coefficient of variation (CV) was equal to 4.1% and 4.8%, with ICC_(3,1)_ of 0.92 and 0.85 for repeated measures from PRET_max_ and RAMP, respectively. Measurement error was 0.15 L·min^-1^ and 2.11 ml·kg^-1^·min^-1^ in PRET_max_ and 0.16 L·min^-1^ and 2.29 ml·kg^-1^·min^-1^ during RAMP for determining absolute and relative $\dot{V}O_{2\mathrm{peak}}$, respectively. The difference in $\dot{V}O_{2\mathrm{peak}}$ between PRET_max_ and RAMP was tending towards statistical significance (26.2 ± 5.1 versus 24.3 ± 4.0 ml·kg^-1^·min^-1^, *P* = 0.055). The 95% LoA were -1.9 ± 4.1 (-9.9 to 6.2) ml·kg^-1^·min^-1^.

**Conclusion:**

The PRET_max_ can be used as a reliable test to measure $\dot{V}O_{2\mathrm{peak}}$ during handcycle exercise in recreationally active participants. Whilst PRET_max_ tended towards significantly greater $\dot{V}O_{2\mathrm{peak}}$ values than RAMP, the difference is smaller than measurement error of determining $\dot{V}O_{2\mathrm{peak}}$ from PRET_max_ and RAMP.

## Introduction

The measurement of peak oxygen uptake ($\dot{V}O_{2\mathrm{peak}}$) is critically important for clinicians, coaches and athletes alike. Within able-bodied participants performing lower-body forms of exercise, not only is it considered to be the best indicator of all-cause mortality [1], but percentage of $\dot{V}O_{2\mathrm{peak}}$ is recommended as a primary measure by which to prescribe exercise intensity tailored to an individual’s fitness, according to the American College of Sports Medicine [2]. Furthermore, $\dot{V}O_{2\mathrm{peak}}$ can be used to evaluate the effects of a training intervention within clinical and athletic populations. Based on the pioneering experiments of Hill and colleagues [3,4] the phenomenon of $\dot{V}O_{2\mathrm{peak}}$ has become evident and has led to the development of methods by which it can be measured. In a contemporary setting, the measurement of $\dot{V}O_{2\mathrm{peak}}$ often takes the form of a ramp-incremented protocol (RAMP), requiring participants to exercise at increasing workloads until volitional exhaustion [5]. However, it is argued that a RAMP test is unnatural, as the open-loop nature of the test means there is no known end-point in terms of exercise time, and it does not allow participants to control pacing strategy or the exercise intensity [6].

The idea to use time-limited exercise stages clamped at specific ratings of perceived exertion (RPE) using the Borg 6–20 RPE Scale [7] during a graded exercise test came from earlier work by Eston and colleagues on cardiac patients [8] and later on young active men [9]. Their research provided initial proof of concept and rationale for a series of studies on the efficacy of perceptually-regulated exercise testing (PRET), with a known end-point RPE, involving different exercise modalities and population groups (see Coquart et al. [10,11] for reviews) as a valid means of predicting $\dot{V}O_{2\mathrm{peak}}$ from the $\dot{V}O_{2}$ at submaximal RPE. Recently, there has been considerable interest in the application of a maximal PRET (PRET_max_), also interchangeably referred to as a self-paced $\dot{V}O_{2\mathrm{peak}}$ test (SPV), to measure $\dot{V}O_{2\mathrm{peak}}$ [12–24]. The original PRET_max_ [18] consisted of the same 2-min, verbally anchored RPE stages (11, 13, 15, 17) as those applied by Eston et al. [25] with the addition of RPE 20 to produce a maximal effort and freedom to change power output (PO) or speed on a moment to moment basis during each of the perceptually-regulated bouts. Other authors have used protocols with seven stages at RPE 8, 10, 12, 14, 16 and 20 [13] and six, 3-min stages at RPE 9, 11, 13, 15, 17 and 20 [14]. As indicated above, these closed-loop protocols have the advantages of known duration and in allowing participants a level of autonomy to control exercise intensity whilst maintaining a fixed RPE.

Though there is an acceptance over the potential use of the PRET_max_, a debate exists over how the $\dot{V}O_{2\mathrm{peak}}$ value measured during PRET_max_ compares to that derived from RAMP testing. Notably, 8% and 5% greater $\dot{V}O_{2\max}$ values were observed from the SPV during cycle ergometry [18] and treadmill running [17], respectively. However, these results have been questioned on the basis that they are confounded either by differences in test duration or use of different ergometers for the RAMP (motorized treadmill) and PRET_max_ (non-motorized treadmill) trials [26]. Also, the small difference in $\dot{V}O_{2\max}$ reported between RAMP and PRET_max_ in the treadmill study [17], despite reporting otherwise, did not exceed the difference which could be attributed to the measurement error of $\dot{V}O_{2\mathrm{peak}}$ in their study. In contrast, no differences in $\dot{V}O_{2\mathrm{peak}}$ from PRET_max_ and RAMP have been observed when using the original [20,21] and variants of the SPV [13,14]. Methodological inconsistencies are further found in a study comparing PRET_max_ and RAMP protocols that have changed incline and speed, respectively [16]. Conversely, a study has also compared the PRET_max_ and RAMP using changes in speed and incline, respectively [19]. In these instances the protocol that altered the incline produced a significantly greater $\dot{V}O_{2\mathrm{peak}}$ In the case of Hogg et al. [16] this was the PRET_max_, whilst for Scheadler and Devor [19] it was RAMP. Blinding of participants offers another example of discrepancy between studies with some blinding participants to either the speed or PO during trials [14,20–22], and others not blinding participants [17,18,23]. The combination of equivocal findings with methodological and statistical analysis discrepancies make it difficult to draw firm conclusions as to the use of the PRET_max_ for determining $\dot{V}O_{2\mathrm{peak}}$ compared to RAMP testing. In addition, only a limited number of studies have assessed the reliability of peak physiological responses to the PRET_max_ [20,22,27]. During RAMP, the day-to-day variation for $\dot{V}O_{2\mathrm{peak}}$ has been characterised as having a coefficient of variation (CV) of 3–4% [28,29]. A CV of 3% [20] and 4.7% [27] in $\dot{V}O_{2\mathrm{peak}}$ have been observed from repeat PRET_max_ trials, although corroborating evidence is required in order to support these results.

Though evidence for the use of the PRET_max_ is developing, results are limited to lower body forms of exercise. Whilst a submaximal PRET using arm crank ergometry has been shown to be valid for the prediction of $\dot{V}O_{2\mathrm{peak}}$ [30], no study has investigated the PRET_max_ using an upper body exercise modality. It has been shown that RPE can be used to regulate exercise intensity during handcycle exercise [31] and wheelchair propulsion using experienced [32] and novice participants [33]. Evidence supporting the ability of the PRET_max_ protocol to measure $\dot{V}O_{2\mathrm{peak}}$ during upper body exercise could have implications for the exercise testing of many people with disabilities, such as spinal cord injury, where exercise choice is limited to those involving the upper body. If the $\dot{V}O_{2\mathrm{peak}}$ from PRET_max_ was shown to be comparable to that measured in RAMP within participants who are novice to upper body exercise, this could support its use in more experienced users, such as those with chronic spinal cord injury or wheelchair sportspersons. As such, this study aimed to compare the $\dot{V}O_{2\mathrm{peak}}$ values obtained from a PRET_max_ and RAMP protocol during handcycling in novice users. Based on previous research it was hypothesised that there would be no difference in $\dot{V}O_{2\mathrm{peak}}$ between PRET_max_ and RAMP and that both would show high reliability [34].

## Methods

### Participants

Twenty (14 male, 6 female), recreationally active able-bodied participants volunteered to take part in this study, which was approved by the Loughborough University Ethics Committee (R15_P067) and conducted in accordance with the Declaration of Helsinki. Written informed consent was obtained from all individual participants included in the study. Descriptive characteristics are presented in Table 1. Participants had no prior experience in handcycling, were free from injury and were not partaking in any regular upper body endurance training, as in Paulson et al. [32].

**Table 1:** Participant descriptive characteristics.

	Males (n = 14)	Females (n = 6)	Group (n = 20)
Age (years)	23 ± 4	22 ± 4	23 ± 4
Height (m)	1.80 ± 0.07	1.64 ± 0.07	1.75 ± 0.10
Body mass (kg)	78.1 ± 13.7	60.6 ± 7.3	72.8 ± 14.5
Physical activity level (h·week^-1^)	6.6 ± 3.1	8.7 ± 3.0	7.1 ± 3.1

Data are presented as mean ± standard deviation (SD).

### Experimental design

Participants completed four trials over a two-week period in a randomised, crossover design. For this, participants completed two RAMP tests in week 1, followed by two PRET_max_ in week 2, or vice versa. $\dot{V}O_{2\mathrm{peak}}$ was determined in a laboratory setting via synchronous handcycle exercise (Invacare Top End Force 3, Elyria, OH, USA) attached to a Cyclus 2 ergometer (Avantronic Richter, Leipzig, Germany). Participants were fitted into the handcycle to feel comfortable but were required to have some elbow flexion at the furthest point in the pedal cycle. Variables that could be changed to achieve the correct fit were distance of the cranks from the backrest and also the angle of the backrest. Measures for handcycle set up were recorded at the first trial and replicated thereafter.

Main trials were separated by a minimum of 48 and a maximum of 120 h. All trials were performed after a 24 h food standardisation period and participants were asked to avoid caffeine and alcohol consumption for six and 24 h, respectively, prior to testing and to not perform any vigorous exercise in the 24 h before testing. In order to standardise nutritional intake and its potential impact on performance, participants recorded their food and drink intake in the 24 h preceding the initial test and replicated this prior to all further trials. To account for diurnal variations of $\dot{V}O_{2}$ and RPE [35], exercise tests were performed at the same time of day within participants.

All testing was conducted by the same investigator (MH), who was not blinded to condition assignment. For all participants the same handcycle was used, as was also the case for the ergometer and breath-by-breath gas analysis system. Prior to all trials participants completed their own self-selected warm-up. Verbal encouragement was provided throughout all trials by the investigator.

### Ramp-incremented $\dot{V}O_{2\mathrm{peak}}$ test (RAMP) with verification phase (VER)

The RAMP started between 20–40 W which was performed for two minutes. The PO was then increased by 20 W·min^-1^ until the participant reached volitional exhaustion or when they were unable to maintain their preferred cadence despite verbal encouragement. Gas exchange variables $\dot{V}O_{2}$, carbon dioxide production ($\dot{V}CO_{2}$) and minute ventilation ($\dot{V}E$) were collected breath-by-breath using an online gas analysis system (Cortex Metalyser 3B, Cortex, Leipzig, Germany), calibrated prior to each use against ambient air, known gas concentrations and a 3 litre calibration syringe (Hans Rudolph Inc., Shawnee, KS, USA), as per the manufacturer’s instructions. Heart rate (HR) was collected via telemetry (Polar RS400, Kempele, Finland). A capillary blood sample from the ear lobe was taken pre and post-test for the determination of blood lactate concentration ([BLa]). Blood was sampled from the ear lobe because of the convenience it provides during upper body exercise. Blood samples were analysed using Biosen C-line monitor (EKF Diagnostics, Barleben, Germany) calibrated prior to use as per manufacturer instructions. Differentiated measures of peripheral (RPE_P_), central (RPE_C_) and overall (RPE_O_) RPE were verbally reported by the participant in the last 15 s of each stage using Borg’s 6–20 RPE scale [7]. Prior to all trials participants were provided with standardised verbal instructions on the use of Borg’s RPE scale [7]. Participants were instructed to maintain their preferred cadence throughout, whilst all data other than cadence and the RPE scale were obscured from view of the participants for the test duration.

A verification phase (VER) was performed in a subset of 11 participants (six male, 5 female; 22 ± 3 years; 69.6 ± 15.5 kg; 1.72 ± 0.10 m) to confirm the $\dot{V}O_{2\mathrm{peak}}$ achieved in RAMP. Following the end of the RAMP participants received 10 min rest where they either performed unloaded handcycle exercise or rested in the handcycle. The VER was performed at PO 5 W greater than the end of the RAMP. Participants continued until they reached volitional exhaustion or until they were unable to maintain their preferred cadence despite verbal encouragement. Inspired and expired air were collected throughout.

### Perceptually-regulated $\dot{V}O_{2\mathrm{peak}}$ test (PRET_max_)

Participants completed five, two-minute stages in a continuous manner where RPE_O_ was clamped and progressively increased each stage [https://dx.doi.org/10.17504/protocols.io.idcca2w, PROTOCOL DOI]. The five stages corresponded to RPE 11 (light), 13 (somewhat hard), 15 (hard), 17 (very hard) and 20 (maximal exertion) on Borg’s RPE scale [7]. The participants were responsible for controlling the PO using 2 buttons attached to one of the handles that either increased or decreased PO by 5 W each time. Throughout each stage participants were instructed to change the PO as often as was required in order to maintain the desired RPE, and in the final stage in order to achieve exhaustion at the end of the stage. Furthermore, participants were asked to maintain their preferred cadence for the test duration and were reminded throughout each stage of the target RPE. As with RAMP testing, all data other than cadence and RPE scale were blinded from the view of the participants in accordance with previous research [14,20–22]. In contrast to RAMP, elapsed time was also visible during PRET_max_ as knowledge of the end point was considered important for pacing. Gas exchange variables, HR and PO were collected throughout the test. Differentiated RPE were collected at the end of each stage. A capillary blood sample was taken pre and immediately post-test for the measurement of [BLa].

### Data processing and statistical analysis

Gas exchange data were cleaned by removing from analysis any data points that lay greater than three standard deviations outside the local 60 s rolling average [36]. For both protocols PO, HR and gas exchange variables were subjected to a 30 s rolling average with the highest single value from throughout the test taken as the peak response. An a-priori power analysis using G*Power 3.1 (Franz Faul, Universitat Kiel, Germany) was conducted to determine appropriate sample size. Given the test-retest analysis on $\dot{V}O_{2\mathrm{peak}}$ in a previous study [37] providing an effect size of 0.97, a sample size of 20 was deemed to provide statistical power of 80% at an alpha of 0.05 for the assessment of difference in $\dot{V}O_{2\mathrm{peak}}$ between protocols. Analysis was performed using IBM SPSS Statistics 22 (SPSS Inc., Chicago, IL.). Parametric data are presented as mean ± standard deviation (SD) whilst non-parametric data are presented as median (interquartile range). Statistical significance was accepted at *P* < 0.05.

All data were checked for normal distribution using the Shapiro-Wilk test statistic. Heteroscedasticity was assessed using the maximal responses from PRET_max_ and RAMP. The absolute difference was correlated against the mean of the two values, with data said to be heteroscedastic if the correlation was significant. Data for HR_peak_ and PO_peak_ were found to be heteroscedastic, however log transformation of data did not improve this, so the original non-transformed data were used for these, and all other variables. Any learning effect via familiarisation with upper body exercise was assessed across trial one to four, independent of protocol, using one-way analysis of variance (ANOVA) and Greenhouse-Geisser epsilon, with Bonferroni post-hoc tests for multiple comparisons. The $\dot{V}O_{2\mathrm{peak}}$ measured in RAMP was confirmed by performing paired samples t-test on values measured in RAMP and VER. Differences in test duration and peak physiological responses between RAMP and PRET_max_ were assessed via paired samples t-test and for maximal perceptual responses using Wilcoxon Signed Rank test. Bland-Altman plots with 95% limits of agreement (LoA) were performed to assess the agreement for peak physiological variables between the two protocols [38]. Paired t-test and 95% LoA were performed on the maximal value for each measure obtained during PRET_max_ and RAMP across repeat trials.

Relative reliability of peak physiological variables was assessed by calculating the coefficient of variation (CV) and intraclass correlation coefficient (ICC_3,1_) using an openly available spreadsheet [39]. ICC_(3,1)_ were interpreted using Munro’s classification where 0–0.25 classed as little to no correlation, 0.26–0.49 low correlation, 0.50–0.69 moderate correlation, 0.70–0.89 high correlation and 0.90–1.00 very high correlation [34]. Absolute measures of reliability were assessed by calculation of the measurement error and reproducibility using the Smallest Detectable Difference (SDD). The measurement error was calculated as the within-subject standard deviation and SDD as 2.77 multiplied by the measurement error [40].

## Results

All participants completed all trials successfully. There was no learning effect or familiarisation evident as no significant differences were found across trial 1 to trial 4 for absolute $\dot{V}O_{2\mathrm{peak}}$ (F_(1.5)_ = 0.668, *P* = 0.477), relative $\dot{V}O_{2\mathrm{peak}}$ (F_(1.5)_ = 0.568, *P* = 0.521), HR_peak_ (F_(1.9)_ = 0.969, *P* = 0.387) or PO_peak_ (F_(1.4)_ = 1.092, *P* = 0.329). Furthermore Bonferroni post-hoc analysis found that there was no difference between any pair of trials for absolute $\dot{V}O_{2\mathrm{peak}}$ (range 1.74 ± 0.46 to 1.82 ± 0.52 L·min^-1^, *P* > 0.669), relative $\dot{V}O_{2\mathrm{peak}}$ (range 23.77 ± 3.96 to 24.91 ± 5.22 ml·kg^-1^·min^-1^, *P* > 0.999), HR_peak_ (range 162 ± 17 to 165 ± 16 beats·min^-1^, *P* > 0.872) or PO_peak_ (range 111 ± 36 to 117 ± 38 W, *P* > 0.075). There was no significant difference in absolute (mean difference, 95% confidence interval; 0.0, -0.1–0.1 L·min^-1^; t_(10)_ = 0.364, *P* = 0.724) or relative (0.1, -1.4–1.7 ml·kg^-1^·min^-1^; t_(10)_ = 0.181, *P* = 0.860) $\dot{V}O_{2\mathrm{peak}}$ between RAMP and VER for the first trial (Table 2). This was also found for the second RAMP trial (0.0, -0.1–0.1 L·min^-1^; t_(10)_ = -0.245, *P* = 0.812 and 0.5, -2.1–1.2 ml·kg^-1^·min^-1^; t_(10)_ = -0.635, *P* = 0.541). Test duration was significantly longer in PRET_max_ than during RAMP (195, 155–235 s; t_(19)_ = 10.307, *P* < 0.005). Descriptive statistics for the maximal responses obtained across repeat trials in both PRET_max_ and RAMP tests are presented in Table 3.

**Table 2:** Descriptive statistics for $\dot{V}O_{2\mathrm{peak}}$ measured during RAMP and VER.

	RAMP	VER	*P* value
Trial 1 $\dot{V}O_{2\mathrm{peak}}$ (L·min^-1^)	1.6 ± 0.5	1.6 ± 0.5	0.724
Trial 1 $\dot{V}O_{2\mathrm{peak}}$ (ml·kg^-1^·min^-1^)	22.6 ± 3.7	22.5 ± 3.6	0.860
Trial 2 $\dot{V}O_{2\mathrm{peak}}$ (L·min^-1^)	1.6 ± 0.6	1.6 ± 0.5	0.812
Trial 2 $\dot{V}O_{2\mathrm{peak}}$ (ml·kg^-1^·min^-1^)	21.9 ± 5.0	22.4 ± 4.2	0.541

Data are presented as mean ± SD. Analysis was performed on a subset of 11 participants from the full cohort of 20. $\dot{V}O_{2\mathrm{peak}}$, peak oxygen uptake.

**Table 3:** Descriptive statistics for peak responses from best trial for each protocol.

	PRET_max_	RAMP	*P* value
$\dot{V}O_{2\mathrm{peak}}$ (L·min^-1^)	1.9 ± 0.5	1.8 ± 0.5	0.140
$\dot{V}O_{2\mathrm{peak}}$ (ml·kg^-1^·min^-1^)	26.2 ± 5.1	24.3 ± 4.0	0.055
HR_peak_ (beats·min^-1^)	168 ± 15^*^	163 ± 17	0.015
[BLa]_peak_ (mmol·L^-1^)	8.57 ± 2.31^*^	7.36 ± 1.87	0.006
RER_peak_	1.38 ± 0.12	1.48 ± 0.14^*^	0.005
$\dot{V}E_{\mathrm{peak}}$ (L·min^-1^)	92.4 ± 35.5	89.1 ± 36.3	0.317
PO_peak_ (W)	110 ± 40	122 ± 34^*^	< 0.005
Duration (s)	600 ± 0^*^	405 ± 85	< 0.005
RPE_P_	20 (20 to 20)^*^	20 (19 to 20)	0.034
RPE_C_	20 (17 to 20)^*^	18 (17 to 20)	0.039
RPE_O_	20 (20 to 20)^*^	19 (18 to 20)	< 0.0005

Data are presented as mean ± SD or median (Inter-Quartile Range, IQR).

*: significant difference between protocols. $\dot{V}O_{2\mathrm{peak}}$, peak oxygen uptake; HR_peak_, peak heart rate; [BLa]_peak_, peak blood lactate concentration; PO_peak_, peak power output; RER_peak_, peak respiratory exchange ratio; $\dot{V}E_{\mathrm{peak}}$, peak minute ventilation; RPEP, RPEC, RPEO, peripheral, central and overall Ratings of Perceived Exertion.

### Agreement between protocols

**Fig 1:**
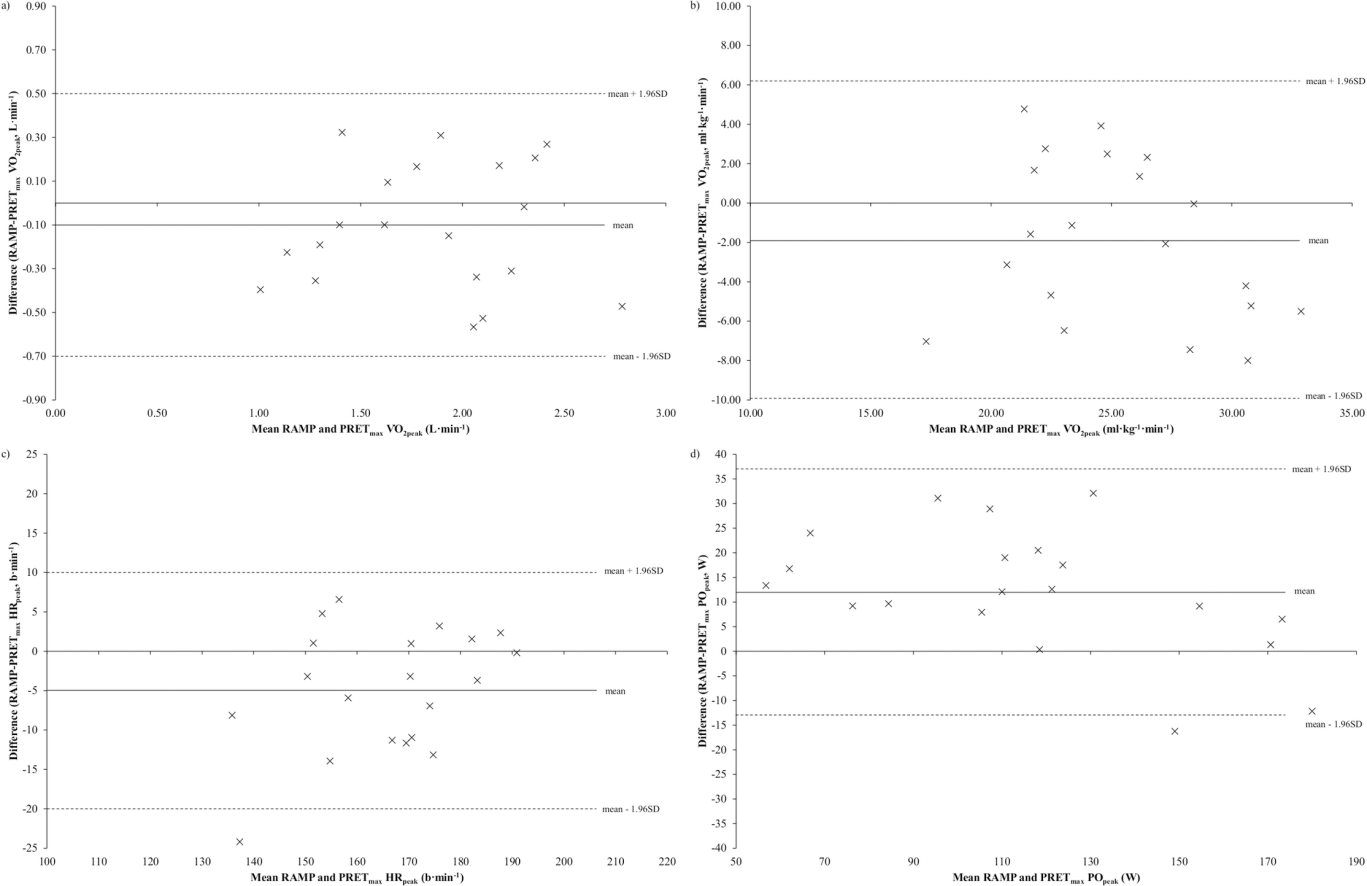
**Bland-Altman plots showing 95% LoA for a) absolute $\dot{V}O_{2\mathrm{peak}}$, b) relative $\dot{V}O_{2\mathrm{peak}}$, c) HR_peak_ and d) PO_peak_.** Mean difference between RAMP and PRET_max_ trials is indicated by solid black line with upper and lower limits indicated by dotted lines.

When using the maximum value across repeat trials for each protocol, PRET_max_ produced significantly greater values for HR_peak_ (5, 1–8 beats·min^-1^; t_(19)_ = 2.668, *P* = 0.015) and [BLa]_peak_ (1.21, 0.39–2.04 mmol·L^-1^; t_(19)_ = 3.075, *P* = 0.006) compared to RAMP. PRET_max_ also resulted in significantly greater peak values for RPE_P_ (Z = -2.212, *P* = 0.034), RPE_C_ (Z = -2.060, *P* = 0.039) and RPE_O_ (Z = -3.482, *P* < 0.005) than RAMP. Conversely, RAMP led to significantly greater values for PO_peak_ (12, 6–18 W; t_(19)_ = 4.278, *P* < 0.005) and RER_peak_ (0.10, 0.03–0.17 L·min^-1^; t_(19)_ = 3.148, *P* = 0.005) than PRET_max_. There was no significant difference in either absolute (-0.1, -0.2–0.0, t_(19)_ = -1.539, *P* = 0.140) and relative (-1.9, -3.4–0.1 ml·kg^-1^·min^-1^, t_(19)_ = -2.041, *P* = 0.055) $\dot{V}O_{2\mathrm{peak}}$, or peak minute ventilation (VE_peak_) (-3.3, -31.7–3.4 L·min^-1^, t_(19)_ = -1.027, *P* = 0.317) between RAMP and PRET_max_. Bland-Altman plots with 95% LoA showing the agreement in absolute and relative $\dot{V}O_{2\mathrm{peak}}$, HR_peak_ and PO_peak_ are displayed in Fig 1.

### Reliability

Test-retest statistics for PRET_max_ and RAMP are shown in Table 4. Measurement error and CV for relative $\dot{V}O_{2\mathrm{peak}}$ were slightly lower for PRET_max_ compared to RAMP, whilst the two protocols had identical measurement error and CV for HR_peak_. For PO_peak_ the measurement error and CV are greater for PRET_max_ compared to RAMP. ICC_(3,1)_ was classified as “very high” for absolute and relative $\dot{V}O_{2\mathrm{peak}}$ during PRET_max_. During RAMP the ICC_(3,1)_ was “very high” for absolute $\dot{V}O_{2\mathrm{peak}}$ and “high” for relative $\dot{V}O_{2\mathrm{peak}}$. For HR_peak_ and PO_peak_ ICC_(3,1)_ were “very high” for both PRET_max_ and RAMP.

**Table 4:** Test-retest reliability statistics for peak physiological variables obtained in PRET_max_ and RAMP protocols.

	PRET_max_				RAMP			
	CV (%)	Measurement error	SDD	ICC_(3,1)_	CV (%)	Measurement error	SDD	ICC_(3,1)_
$\dot{V}O_{2\mathrm{peak}}$ (L·min^-1^)	4.2	0.15	0.42	0.96	4.4	0.16	0.44	0.96
$\dot{V}O_{2\mathrm{peak}}$ (ml·kg^-1^·min^-1^)	4.1	2.11	5.86	0.92	4.8	2.29	6.34	0.85
HR_peak_ (beats·min^-1^)	2.0	6	17	0.93	2.0	6	17	0.94
[BLa]_peak_ (mmol·L^-1^)	7.0	1.17	3.23	0.89	8.4	1.13	3.14	0.82
PO_peak_ (W)	5.1	11	29	0.97	1.9	4	12	0.99
RER_peak_	4.4	0.11	0.31	0.59	4.5	0.12	0.34	0.60
VE_peak_ (L·min^-1^)	6.8	11.6	32.2	0.95	8.2	13.1	36.3	0.94

CV, coefficient of variation; SDD, smallest detectable difference; $\dot{V}O_{2\mathrm{peak}}$, peak oxygen uptake; HR_peak_, peak heart rate; [BLa]_peak_, peak blood lactate concentration; PO_peak_, peak power output; RER_peak_, peak respiratory exchange ratio; VE_peak_, peak minute ventilation

## Discussion

The purpose of this study was to assess the ability of a PRET_max_ to quantify $\dot{V}O_{2\mathrm{peak}}$ during handcycle exercise in novice users and also to compare the $\dot{V}O_{2\mathrm{peak}}$ measured between PRET_max_ and RAMP. A further aim was to investigate the test-retest reliability of the PRET_max_ and RAMP for measuring $\dot{V}O_{2\mathrm{peak}}$. Whilst the $\dot{V}O_{2\mathrm{peak}}$ produced in PRET_max_ (26.2 ± 5.1 ml·kg^-1^·min^-1^) was, in statistical terms, tending towards being significantly greater than that found in RAMP (24.3 ± 4.0 ml·kg^-1^·min^-1^), the mean difference in $\dot{V}O_{2\mathrm{peak}}$ (1.9 ml·kg^-1^·min^-1^) found between protocols is smaller than the measurement error for determining $\dot{V}O_{2\mathrm{peak}}$ from both PRET_max_ and RAMP (2.1 and 2.3 ml·kg^-1^·min^-1^, respectively). Whilst recognising this small difference in $\dot{V}O_{2\mathrm{peak}}$ between the protocols has minimal physiological relevance, we also believe it cannot be considered to reflect a systematic difference in $\dot{V}O_{2\mathrm{peak}}$ between protocols, as it did not exceed the measurement error observed within each of the two test protocols. Furthermore, the difference in absolute $\dot{V}O_{2\mathrm{peak}}$ between the two protocols was not approaching statistical significance. For evidence of a systematic difference, one would expect a similar statistical difference in both relative and absolute measures of $\dot{V}O_{2\mathrm{peak}}$ [23,27]. Conspicuously other studies showing an increased relative $\dot{V}O_{2\mathrm{peak}}$ during PRET_max_ have not reported the accompanying absolute values [16–18]. As such, this supports the use of the PRET_max_ as a reliable protocol to measure $\dot{V}O_{2\mathrm{peak}}$ in this population and that it provides comparable values to that measured during RAMP.

Previously Straub et al. [20] found CV for $\dot{V}O_{2\mathrm{peak}}$ of 4% for RAMP and 3% for PRET_max_, whilst Jenkins et al. [27] report values of 4.7% and 8.2% for healthy individuals and cardiac rehabilitation patients, respectively. Corresponding results of 4.8% for RAMP and 4.1% for PRET_max_ in the present study support the reliability of the PRET_max_. Furthermore, measurement error of both RAMP and PRET_max_ have been reported to be 0.13 L·min^-1^ [20], with this study resulting in measurement error of 0.16 and 0.15 L·min^-1^ for RAMP and PRET_max_, respectively. Whilst the CV and measurement error reported for $\dot{V}O_{2\mathrm{peak}}$ from PRET_max_ appear to be slightly greater than previously identified, the current study utilised participants that were unfamiliar with handcycle exercise whereas previously trained cyclists were used [20]. This suggests that a reliable measurement of $\dot{V}O_{2\mathrm{peak}}$ can be made using the PRET_max_ even in novice users. In addition, the current results help corroborate findings from previous research [20] as to the reliability of identifying PO_peak_, HR_peak_, RER_peak_ and [BLa]_peak_ from PRET_max_.

Whilst the current results support the use of the PRET_max_ as comparable to RAMP for quantifying $\dot{V}O_{2\mathrm{peak}}$, research has thus far been equivocal. Previous studies have reported both an increase [12,16–18,23] and no difference [13–15,20–23] in $\dot{V}O_{2\mathrm{peak}}$ with PRET_max_ compared to RAMP. Though theoretically these results do provide for an interesting discussion as to the merits of the PRET_max_ and RAMP, there are methodological differences in studies, particularly around the implementation of the final RPE 20 stage which make synthesis of findings difficult. In proposing the SPV test, Mauger and Sculthorpe acknowledged that the protocol design “allows subjects to self-pace their work rates according to a given end point” [18], p. 59]. However in a subsequent study they instructed participants to “vary their speed to match the RPE for each given moment, rather than to pace themselves according to the projected end point of the test” [17], p. 1213], in direct conflict with their initial instruction. This method results in an immediate premature sprint with a rapid increase in power output, followed immediately by diminishing speed or PO to the end-point of the test [16,18]. The conflicting instructions and apparently diverse methodology in the two studies [17,18] may account for differences in the application of the pacing strategy applied in SPV studies. Furthermore, in the study of Astorino et al. [12] it would seem that little instruction was given on how to conduct their SPV as evidenced by the differences in the pacing strategy used by participants, particularly at RPE 20. This is highlighted by participants having to stop before the test had finished (mean test duration was 9.6 ± 0.8 min for a test designed to have five, two-minute stages).

In contrast, this study along with others [13,14,20,21] instructed participants to change the PO as often as was required in order to maintain the desired RPE and in the final stage such that exhaustion occurred at the end of the stage. This implementation of the PRET_max_ allows true self-pacing to the end point throughout the test and has consistently been shown to produce $\dot{V}O_{2\mathrm{peak}}$ values that agree with those obtained from RAMP [13,14,20,21]. Though in fact recent research would suggest that the pacing strategy used has no influence on the $\dot{V}O_{2\mathrm{peak}}$ [41] The mean difference in $\dot{V}O_{2\mathrm{peak}}$ between protocols has previously been reported as 0.002 L·min^-1^ [20], 0.05 L·min^-1^ [13], -0.8 ml·kg^-1^·min^-1^ [14], 0.04 L·min^-1^ and 0.13 ml·kg^-1^·min^-1^ [23], with corresponding values of 0.1 L·min^-1^ and 1.9 ml·kg^-1^·min^-1^ in this study. Though greater than in previous research and potentially suggestive of reduced agreement in $\dot{V}O_{2\mathrm{peak}}$ between PRET_max_ and RAMP during handcycle exercise, the observed 95% LoA serve to corroborate those of previous research. In finding no significant difference in $\dot{V}O_{2\mathrm{peak}}$ between PRET_max_ and RAMP, Faulkner et al. [15], reported mean difference for $\dot{V}O_{2\mathrm{peak}}$ of 3.0 (lower to upper 95% limits, -8.5 to 14.5) ml·kg^-1^·min^-1^, with equivalent values of -1.9 (-9.9 to 6.2) ml·kg^-1^·min^-1^ in the current study. The 95% LoA are a better measure of agreement than the mean difference as they factor both the systematic and random variance between protocols [38]. As such, the PRET_max_ is shown to be comparable to RAMP for measurement of $\dot{V}O_{2\mathrm{peak}}$ during handcycle exercise.

Mechanisms have been proposed to explain the phenomenon of increased $\dot{V}O_{2\mathrm{peak}}$ due to PRET_max_, but these appear to have no scientific underpinning. An increased extraction of oxygen at the muscle due to altered muscle recruitment or limb blood flow has been proposed [17], whilst evidence has questioned the physiological possibility of this occurrence [42]. Increased cardiac output during PRET_max_ has also been proposed as a mechanism for increased $\dot{V}O_{2\mathrm{peak}}$ [12,23]. Astorino et al. [12] posit that a decreased physiological load in submaximal self-paced exercise, in comparison to prescribed intensities [43], minimised fatigue in the early stages of the PRET_max_ to allow a greater “end spurt” in the final stage, leading to an increased cardiac output and $\dot{V}O_{2\max}$. Though increased cardiac output during PRET_max_ most certainly offers an interesting perspective, attribution of this end spurt and increased cardiac load to the Central Governor Theory [44] seems to contradict the premise of a controller that serves to regulate work rate in order to avoid catastrophic disturbances to homeostasis. Moreover, the existence of a greater $\dot{V}O_{2\mathrm{peak}}$ due to an end spurt or an “all out” effort in the final RPE20 stage [12,16–18,23] is challenged by findings of similar $\dot{V}O_{2\max}$ values between a RAMP test and a three minute all-out protocol [13,45]. Jenkins et al. [23] also attributed the higher $\dot{V}O_{2\mathrm{peak}}$ observed in their study to an increased cardiac output in the SPV. However, their finding can be questioned based on the significantly greater arteriovenous oxygen difference (a-vO_2_ diff) reported in RAMP [23]. When calculating the expected $\dot{V}O_{2\mathrm{peak}}$ from the product of cardiac output and a-vO_2_ diff, in accordance with the Fick principle, there is no difference in the $\dot{V}O_{2\mathrm{peak}}$ between protocols (both 4.23 L·min^-1^). This value is also greater than the reported measured maximal values for RAMP (3.34 ± 0.88 L·min^-1^) and SPV (3.45 ± 0.87 L·min^-1^) [23]. These discrepancies in data among other methodological issues in the study of Jenkins et al. [23] have drawn strong criticism [46,47]. At present, the lack of evidence for a mechanism leading to increased $\dot{V}O_{2\mathrm{peak}}$ with PRET_max_, as well as corroborating evidence showing no difference with RAMP lends support towards the PRET_max_ being a reliable measure of $\dot{V}O_{2\mathrm{peak}}$ and comparable to RAMP.

Though the current finding of comparable measurement of $\dot{V}O_{2\mathrm{peak}}$ PRET_max_ and RAMP during handcycle exercise adds to a growing body of evidence, our results show significantly increased PO_peak_ in RAMP compared to PRET_max_. Investigations using lower limb, as opposed to upper limb cycling, report no difference in PO_peak_ between PRET_max_ and RAMP [13,20] and an increase in PO_peak_ with PRET_max_ [14,18]. The increased PO_peak_ with RAMP, but trend towards greater $\dot{V}O_{2\mathrm{peak}}$ with PRET_max_ initially appears to suggest a dissociation between the two variables. However, it is more likely that the increased PO_peak_ and RER_peak_ in RAMP is, at least partly, attributable to the ramp rate used in this study. An increased ramp rate, 12 W·min^-1^ versus 6 W·min^-1^ has been shown to lead to increased PO_peak_ (168 ± 28 versus 149 ± 26 W, *P* < 0.001) and RER_peak_ (1.17 ± 0.07 versus 1.11 ± 0.06, *P* = 0.001), with no difference found in $\dot{V}O_{2\mathrm{peak}}$ (3.06 ± 0.65 versus 2.96 ± 0.48 L·min^-1^, *P* = 0.270) during arm crank ergometry [48]. With an increase in mechanical work there is a lag in the $\dot{V}O_{2}$ response, which is accentuated by faster ramp rates [49] and leads to similar $\dot{V}O_{2\mathrm{peak}}$ values being achieved with greater PO_peak_. It is likely that the ramp rate used in this study elevated the PO_peak_ and limits the ability to compare the PO_peak_ obtained from PRET_max_ with that from RAMP. This is a limitation of this study and investigation of the PRET_max_ against a RAMP with a slower ramp rate during handcycle exercise is warranted.

### Methodological considerations

This study supports the use of the PRET_max_ for the measurement of $\dot{V}O_{2\mathrm{peak}}$ during handcycle exercise. A benefit, as previously noted, of the use of the PRET_max_ allows the participant to begin the test knowing how long they have to exercise for, which is not evident in RAMP testing. Furthermore as the workload is set by the participant, the need to find an appropriate starting PO and PO increment, a potential limitation of the RAMP, is removed. However, a limitation of the current study is that it only supports the use of the PRET_max_ to measure $\dot{V}O_{2\mathrm{peak}}$. Whilst RAMP testing during upper body exercise allows the calculation of physiological thresholds related to exercise intensity classification [50,51], the same is not known for the PRET_max_. Therefore if such variables are considered an important outcome of an exercise test then this must be factored in when choosing a testing protocol until the calculation of thresholds during PRET_max_ has been shown.

## Conclusion

In conclusion, this is the first study to show that PRET_max_ can be used as a valid and reliable protocol to measure $\dot{V}O_{2\mathrm{peak}}$ during handcycle exercise in novice users. As such, both hypotheses can be accepted. Though due to the demographics of the participants, the results can only be applied to young, recreationally active, able-bodied participants. Supplementary investigations are warranted to determine the suitability of the use of the PRET_max_ during handcycle exercise for other population groups.

## Supporting information

S1 File Descriptive data and raw data from each trial. (XLSX)
